# Disseminated Histoplasmosis Masquerading as Metastatic Malignancy: A Diagnostic Odyssey in an Immunocompromised Host

**DOI:** 10.7759/cureus.112651

**Published:** 2026-07-14

**Authors:** Aninda B Chanda, Atia Zaka-Ur-Rab, Imad Ali, Kartik Varshney, Asjad A Siddiqui

**Affiliations:** 1 Department of Surgery, Jawaharlal Nehru Medical College & Hospital, Aligarh Muslim University, Aligarh, IND

**Keywords:** bilateral adrenal masses, core needle biopsy, disseminated fungal infection, hepatitis c, histoplasmosis, immunocompromised host, india, paraumbilical nodule

## Abstract

Disseminated histoplasmosis is a rare systemic fungal infection caused by *Histoplasma capsulatum*, typically reported in immunocompromised individuals. Bilateral adrenal involvement, though a recognized but uncommon manifestation, frequently presents a formidable diagnostic challenge owing to its striking radiologic resemblance to disseminated malignancy. We report a case of a 46-year-old male farmer from India presenting with abdominal pain, a progressive paraumbilical lump, low-grade fever, and weight loss. Contrast-enhanced CT revealed large bilateral adrenal masses, widespread abdominal soft tissue deposits, and hypodense splenic lesions. Although these appearances most closely favored disseminated malignancy of unknown primary, they were nonspecific, and a disseminated infective etiology was retained within the radiologic differential. The patient had an untreated asymptomatic hepatitis C virus (HCV) infection and type 2 diabetes mellitus. Fine-needle aspiration cytology (FNAC) of the bilateral adrenal masses and a core-needle biopsy of the paraumbilical nodule, with histopathological examination using periodic acid-Schiff (PAS) staining, revealed intracellular and extracellular fungal forms with narrow-based budding morphology, consistent with *H. capsulatum*. Morning serum cortisol was preserved at 10.80 µg/dL despite massive bilateral adrenal infiltration. Urine *Histoplasma* galactomannan antigen was negative, underscoring the limitations of serologic tests in non-HIV-associated immunosuppression. This case suggests, rather than establishes, that non-HIV-associated immunosuppression from HCV infection and diabetes mellitus may act as predisposing substrates for invasive fungal disease; a causal role cannot be inferred from a single report and warrants confirmation in larger series.

## Introduction

*Histoplasma capsulatum* is a thermally dimorphic fungus with global distribution, increasingly recognized in the Indian subcontinent, particularly along the Gangetic Plains, though still significantly underdiagnosed due to limited clinical awareness and fungal culture facilities [1,2]. Agricultural workers with prolonged soil exposure constitute a well-established high-risk group [3]. Disseminated histoplasmosis occurs predominantly in immunocompromised hosts. While human immunodeficiency virus (HIV) infection remains the classic predisposing factor, hepatitis C virus (HCV) infection, diabetes mellitus, and hematologic malignancies are increasingly recognized as important substrates [4,5]. Bilateral adrenal involvement of *H. capsulatum* occurs in up to 80% of disseminated cases and closely mimics metastatic malignancy [6,7]. Bilateral adrenal enlargement carries a broad differential that includes bilateral metastases, primary adrenocortical carcinoma, lymphoma, pheochromocytoma, tuberculosis, and histoplasmosis because these entities are radiologically indistinguishable yet demand entirely different management. Tissue biopsy remains the cornerstone of definitive diagnosis, particularly because urine Histoplasma antigen testing yields false-negative results in a significant proportion of non-HIV-associated immunosuppressed patients [8].

We report this case to highlight the diagnostic overlap between disseminated histoplasmosis and metastatic malignancy, the co-occurrence of dual non-HIV-associated immunosuppression, preserved adrenal function despite massive bilateral infiltration, a false-negative urine Histoplasma antigen test, and the indispensability of tissue biopsy for definitive diagnosis.

## Case presentation

A 46-year-old male farmer presented with a two-month history of intermittent, mild-to-moderate, nonradiating diffuse abdominal pain, partially relieved with oral analgesics. This was associated with a one-month history of a progressively enlarging paraumbilical lump, intermittent low-grade fever (maximum 38.0°C) subsiding spontaneously, and significant unintentional weight loss over the preceding two months, subjectively quantified by the patient as progressive loosening of his clothing. The patient was HCV-seropositive and was found to have untreated type 2 diabetes mellitus (T2DM), discovered incidentally during this admission.

Physical examination revealed a firm, non-tender, superficial extra-abdominal lump measuring 2 × 2 cm in the left paraumbilical region. The remainder of the systemic examination was within normal limits. Laboratory evaluation revealed normocytic normochromic anemia, mild thrombocytopenia, and a neutrophil-predominant differential leukocyte count. Biochemical analysis demonstrated hyperglycemia with suboptimal glycemic control and mild transaminitis. The anti-HCV antibody was strongly reactive, whereas the morning serum cortisol remained within the normal reference range, confirming preserved adrenal function despite massive bilateral adrenal infiltration. The complete laboratory profile is summarized in Table 1.

**Table 1:** Summary of laboratory investigations HbA1c, glycated hemoglobin; AST, aspartate aminotransferase; ALT, alanine aminotransferase; EIA, enzyme immunoassay; ECLIA. electrochemiluminescence immunoassay

Investigation	Result	Reference range	Flag
Hemoglobin	11.4 g/dL	13.0-17.0 g/dL	Low
Total leukocyte count	7.2 × 10³/µL	4.0-11.0 × 10³/µL	Normal
Neutrophils (differential)	70.2%	40-75%	Normal
Platelet count	92 × 10³/µL	150-410 × 10³/µL	Low
Random blood glucose	152 mg/dL	<140 mg/dL	High
HbA1c	7.2%	<5.7% (non-diabetic); target <7.0% in diabetes	High
AST	67.8 U/L	5-40 U/L	High
ALT	97.6 U/L	7-56 U/L	High
Blood urea	26 mg/dL	15-40 mg/dL	Normal
Serum creatinine	1.1 mg/dL	0.7-1.3 mg/dL	Normal
Sodium (Na⁺)	142 mmol/L	135-145 mmol/L	Normal
Potassium (K⁺)	4.2 mmol/L	3.5-5.1 mmol/L	Normal
Morning serum cortisol (08:00 h)	10.80 µg/dL	6.0-23.0 µg/dL	Normal
Anti-hepatitis C virus antibody (ECLIA), cutoff index	35.26	<1.0 (non-reactive)	Reactive
Histoplasma galactomannan antigen, urine (EIA)	0.29 EIA units	<1.0 (negative)	Negative

Chest radiography demonstrated bilateral lower-zone haziness with increased bronchovascular markings but no frank consolidation, cavitation, or mediastinal lymphadenopathy. Ultrasonography of the abdomen demonstrated a heterogeneous, predominantly isoechoic lesion in the right suprarenal region measuring approximately 30 × 65 mm and a relatively homogeneous, hyperechoic, well-defined lesion in the left suprarenal region measuring 35 × 40 mm, initially raising suspicion for an adrenal neoplasm. Multiple target lesions were identified diffusely involving all poles of the spleen (largest 12 × 13 mm), and heterogeneous, hyperechoic, well-defined nodular lesions were noted in the intramuscular plane of the right paraumbilical region.

**Figure 1:**
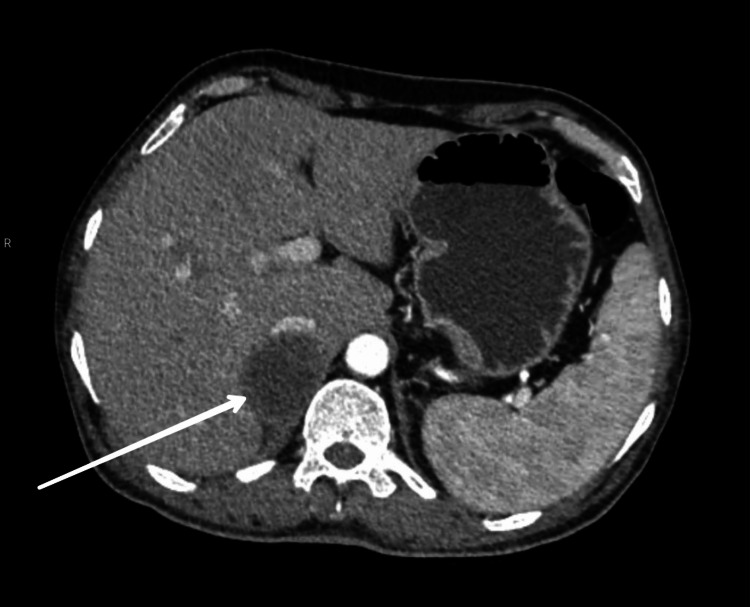
Axial contrast-enhanced CT image of the abdomen demonstrating a large, heterogeneous, soft-tissue-density right suprarenal mass (~6.3 × 4.3 × 9.3 cm; white arrow) infiltrating the adjacent liver parenchyma

Contrast-enhanced computed tomography (CECT) of the abdomen demonstrated large, well-defined, heterogeneous soft-tissue-density lesions involving the bilateral adrenal glands. The right suprarenal lesion measured 6.3 × 4.3 × 9.3 cm, extending from T10-L1, and anterolaterally infiltrating the adjacent liver parenchyma with possible intrahepatic inferior vena cava (IVC) compression (Figure 1).

**Figure 2:**
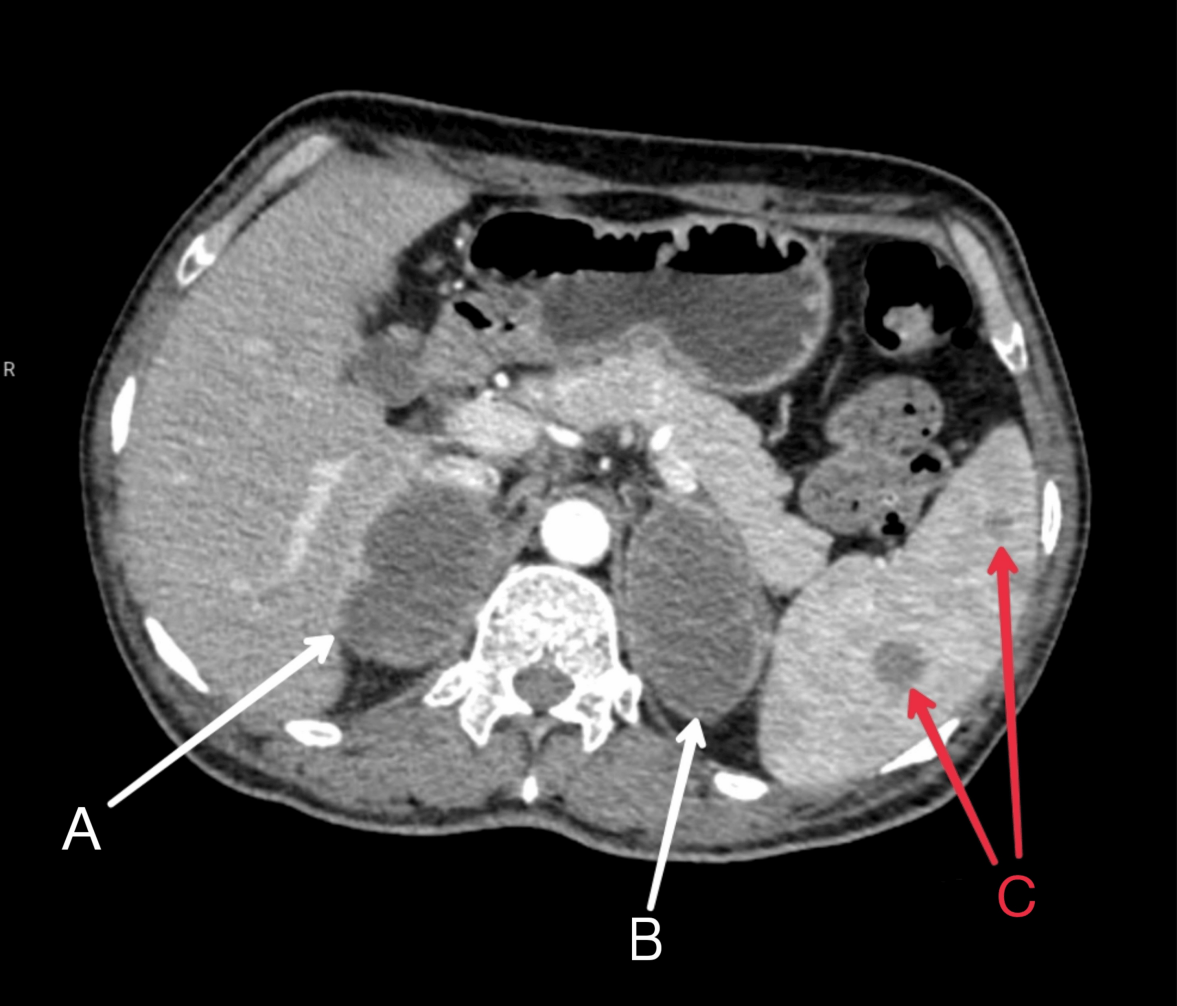
Axial contrast-enhanced CT images of the abdomen demonstrating (A) a heterogeneous right suprarenal mass (~6.3 × 4.3 × 9.3 cm; white arrow), (B) a heterogeneous left suprarenal mass (~6.8 × 4.2 × 9.8 cm; white arrow), and (C) multiple hypodense lesions involving all poles of the splenic parenchyma (red arrows)

The left suprarenal lesion measured 6.8 × 4.2 × 9.8 cm, extending from T11-L1, abutting the tail of the pancreas and the superior pole of the spleen, with maintained fat planes. Multiple well-defined hypodense splenic lesions involving all poles (largest 16 × 13 mm) were also demonstrated (Figure 2).

Additional CT findings by anatomic site included the following: a 17 × 18 × 16.7 mm nodular soft-tissue lesion in the preperitoneal space abutting the rectus muscle (L5-S1 level), a 7.8 × 9.5 mm nodular lesion in intraperitoneal infraumbilical region posterior to the ileal loops, the largest lesion measuring 14 × 22 mm in the left paraumbilical anterior abdominal wall (subcutaneous), an 18 × 25 mm lesion in infraumbilical anterior abdominal wall (subcutaneous) and a 10.5 × 10 mm deposit in the right inguinal region (subcutaneous).

**Figure 3:**
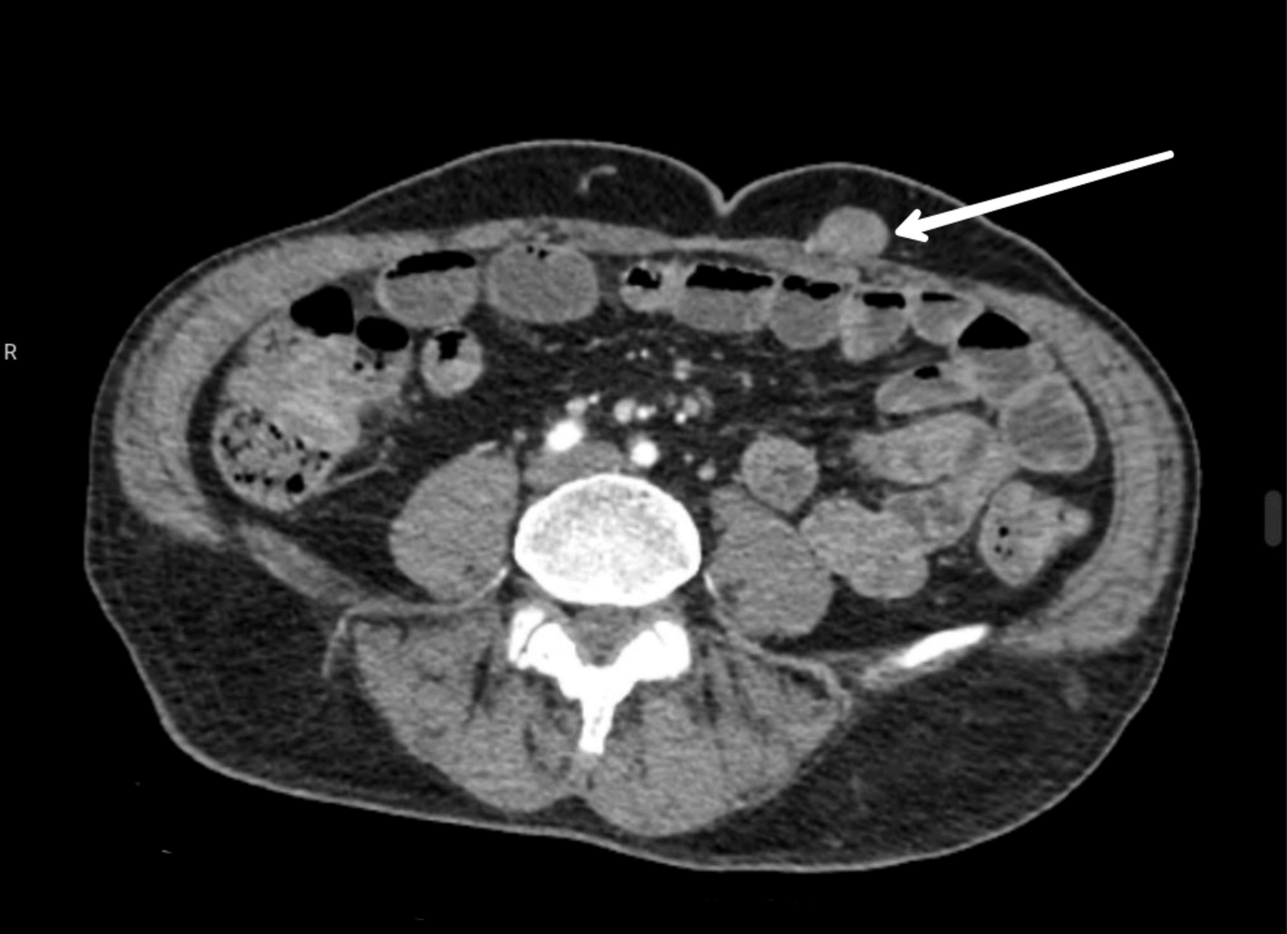
Axial contrast-enhanced CT image of the lower abdomen demonstrating a soft-tissue-density nodular lesion (white arrow) within the deep subcutaneous tissue of the left anterior abdominal wall, along the anterior aspect of the rectus sheath, from which a core-needle biopsy yielded the definitive histopathological diagnosis

The radiologic impression was of large bilateral adrenal masses with extensive peritoneal and subcutaneous deposits, with the primary differential diagnosis being disseminated malignancy; however, a disseminated fungal or tuberculous etiology was also considered in the context of immunosuppression. The left paraumbilical anterior abdominal wall lesion is demonstrated in Figure 3; this lesion was subsequently subjected to core-needle biopsy, yielding the definitive histopathological diagnosis.

CT-guided fine-needle aspiration cytology (FNAC) of the bilateral adrenal masses revealed fungal spores. Core-needle biopsy (three cores) of the paraumbilical nodule demonstrated fibrocollagenous stroma with aggregates of histiocytes, foamy macrophages, and chronic inflammatory infiltrate. Numerous intracellular and extracellular small round-to-oval fungal forms were identified, demonstrating characteristic narrow-based budding morphology. Large areas of necrosis were noted. Periodic acid-Schiff (PAS) staining highlighted the capsule of the fungal organisms. The histomorphologic features were reported as consistent with histoplasmosis.

Fungal blood culture yielded no growth. Urine Histoplasma galactomannan antigen detection by enzyme immunoassay (EIA) yielded a result of 0.29 EIA units (negative; cutoff <1), underscoring the limitations of serologic testing in non-HIV-associated immunosuppression.

In accordance with published guidelines from the Infectious Diseases Society of America (IDSA), the patient was started on liposomal amphotericin B (3 mg/kg/day intravenously) for two weeks, followed by oral itraconazole for 12 months. At the two-month follow-up visit, the patient reported significant symptomatic improvement and was advised to continue follow-up with repeat radiologic imaging to monitor for interval reduction in lesion size.

## Discussion

Our patient, a farmer with 25 years of intensive agricultural soil exposure in wheat and rice fields, fits the classic epidemiologic profile. Exposure to *Histoplasma*-contaminated soil during tillage, particularly in warm, humid climates, constitutes a well-established route of inhalation of infective microconidia [3,9]. *H. capsulatum* infection in India is increasingly recognized as an underreported entity, with the majority of cases documented from eastern and northeastern states, as well as the Gangetic Plains [1,10].

Chronic HCV infection is associated with impaired Th1 cellular immunity and diabetes mellitus with reduced neutrophil and macrophage fungicidal activity; both are recognized risk factors for invasive fungal disease. In our patient, we hypothesize, but cannot prove, that the coexistence of these two non-HIV-associated immunosuppressive states created a permissive environment for fungal dissemination in the absence of HIV or iatrogenic immunosuppression [4,11]. Furthermore, diabetes mellitus is itself a risk factor for disseminated infection because it predisposes to impaired neutrophil and macrophage fungicidal activity [12]. The synergistic immunosuppression from HCV-related hepatic dysfunction and diabetes mellitus in our patient likely created a permissive environment for progressive fungal dissemination in the absence of HIV or exogenous immunosuppressant exposure. Initial CECT of the abdomen suggested disseminated malignancy; however, histopathological examination (HPE) of the biopsy specimen revealed characteristic budding yeast forms, establishing the diagnosis of histoplasmosis. Adrenal histoplasmosis is well recognized to masquerade as malignancy on clinicoradiologic evaluation. Both conditions can produce large, heterogeneous, necrotic-appearing bilateral adrenal masses with involvement of adjacent structures, peritoneal seeding, and splenic deposits, rendering radiologic differentiation unreliable.

Several published case series have demonstrated that adrenal histoplasmosis on CECT is indistinguishable from primary adrenal carcinoma, bilateral adrenal metastases, or lymphoma without histopathological confirmation [6,8]. Fluorodeoxyglucose-positron emission tomography (FDG-PET) similarly demonstrates avid uptake in adrenal histoplasmosis, further compounding the diagnostic confusion [13]. This underscores the irreplaceable role of tissue biopsy in the evaluation of bilateral adrenal masses. In our patient, biopsy of the paraumbilical nodule, a superficial, accessible lesion, provided the definitive diagnosis and obviated the need for the more invasive adrenal biopsy. This pragmatic approach of targeting peripheral, accessible lesions whenever available should be adopted as a standard surgical consideration in similar clinical scenarios.

Adrenal insufficiency (Addison's disease) is a well-recognized complication of bilateral adrenal histoplasmosis, particularly when involvement exceeds 90% of the glandular parenchyma [6,14]. In our patient, morning serum cortisol was 10.80 µg/dL, within the normal reference range of 3.70-19.40 µg/dL, confirming preserved adrenal function despite massive bilateral adrenal infiltration. Preservation of adrenal function despite extensive bilateral involvement has been previously reported and may be explained by the relative sparing of the zona glomerulosa, the zone critical for mineralocorticoid synthesis [15]. Clinicians managing bilateral adrenal histoplasmosis should routinely perform cortisol assessment both to guide steroid supplementation and avoid erroneous attribution of electrolyte abnormalities to adrenal insufficiency.

In our patient, the urine* Histoplasma* antigen test was negative (0.29 EIA units) despite histopathologically confirmed disseminated disease, consistent with previously published cases of antigen-negative histoplasmosis in non-HIV-associated immunosuppressed patients in India [2,16]. This case reinforces that a negative urine *Histoplasma* antigen test does not exclude the diagnosis, and tissue biopsy with appropriate fungal staining, including PAS and Gomori methenamine silver (GMS) staining, remains indispensable for definitive diagnosis.

## Conclusions

Clinicians practicing in agricultural and endemic regions of South Asia should maintain a high index of suspicion for disseminated histoplasmosis in immunocompromised patients presenting with bilateral adrenal masses. Bilateral adrenal masses with widespread peritoneal and subcutaneous deposits, though overwhelmingly suggestive of metastatic malignancy on CECT, mandate histopathological confirmation before oncological labelling. Core needle biopsy of the most accessible peripheral lesion offers a safe, minimally invasive, and diagnostically definitive strategy.
